# Prognostic Factors of Advanced Ovarian Cancer in the Era of HIPEC: A Multicenter Retrospective Study from an ESGO-Certified Center and an ESPSO-Certified Center

**DOI:** 10.3390/biomedicines14020431

**Published:** 2026-02-13

**Authors:** Dimitrios Tsolakidis, Dimitrios Zouzoulas, Dimitrios Kyziridis, Apostolos Kalakonas, Kimon Chatzistamatiou, Vasilis Theodoulidis, Eleni Timotheadou, Antonios-Apostolos Tentes

**Affiliations:** 1 1st Department of Obstetrics & Gynecology, Aristotle University of Thessaloniki, “Papageorgiou” Hospital, 56403 Thessaloniki, Greece; 2 Surgical Oncology Department, Peritoneal Surface Malignancy Program, Kyanous Stavros, 54636 Thessaloniki, Greece; 3 Department of Oncology, Aristotle University of Thessaloniki, “Papageorgiou” Hospital, 56403 Thessaloniki, Greece

**Keywords:** prognostic factors, advanced ovarian cancer, HIPEC

## Abstract

**Background/Objectives**: Cytoreductive surgery (CRS) combined with hyperthermic intraperitoneal chemotherapy (HIPEC) may modify the prognostic impact of established clinical and surgical factors in advanced ovarian cancer. A retrospective study was conducted to identify independent predictors of survival, recurrence patterns and major postoperative complications in the HIPEC setting. **Methods**: In total, 265 women with advanced-stage ovarian cancer operated on between 2015 and 2019 were included. Patients were treated with CRS, with or without HIPEC. Patients’ characteristics, oncological and follow-up information were collected. **Results**: In total, 62.3% underwent primary CRS, and 39.2% received HIPEC, with complete or near-complete cytoreduction (CC-0/1) achieved in 85.6%. Major complications (≥grade III) were recorded in 16.6% of the patients. HIPEC, high peritoneal cancer index (PCI), and greater intraoperative blood loss were found to independently increase the odds of major postoperative complications, while prior surgery was the only independent predictor of local-regional recurrence. The median follow-up was 34 months, with a median progression-free (PFS) and overall survival (OS) of 26 and 77 months, respectively. Multivariable analysis identified systematic lymphadenectomy and serous histology as independent predictors for PFS and also for OS, with the addition of CC-3. Survival analysis with Kaplan–Meier curves revealed that CRS plus HIPEC was associated with significantly better OS, but not PFS, compared with CRS alone. **Conclusions**: Systematic lymphadenectomy, serous histology, and absence of macroscopic gross residual disease emerge as key independent favorable prognostic factors in the HIPEC era, while prior surgery adversely affects loco-regional control. CRS plus HIPEC improved OS in this specific regimen’s perfusion protocol but was associated with higher major postoperative complications, underscoring the need for careful patient selection.

## 1. Introduction

Ovarian cancer is the third most common malignancy in female patients, with an estimated 20,890 new cases in 2025 in the USA [1]. It also remains the leading cause of death for women with breast or genital tract cancers, with an estimated 12,730 deaths and a 51% relative survival rate for 2025 in the USA [1]. Due to its nonspecific and subtle symptoms, it is usually diagnosed at an advanced stage of disease, leading to poor survival rates [2]. Type I epithelial ovarian cancers are generally considered indolent and genetically stable tumors that arise from well-defined precursor lesions, including endometriosis and borderline tumors with low malignant potential. In contrast, type II epithelial ovarian cancers are characterized by intrinsic biological aggressiveness, exhibiting a marked propensity for early dissemination, even when originating from small-volume primary lesions. High-grade serous carcinoma, the most common subtype of epithelial ovarian cancer (accounting for approximately 75% of cases), follows the type II pathway and is typically associated with TP53 mutations and frequent BRCA1/2 alterations [3].

Over the years, efforts have been made to better understand the disease and identify prognostic factors to optimize the primary treatment plan for these patients. The technologies of proteomics, such as mass spectrometry and protein array analysis, have advanced the dissection of the underlying molecular signaling events and revealed the proteomic characterization of ovarian cancer. Proteomics analysis of ovarian cancers, as well as their adaptive responses to therapy, can uncover new therapeutic choices, which can reduce the emergence of drug resistance and potentially improve patient outcomes [4]. The extent of residual tumor has been identified as the most significant prognostic factor since the 1990s [5]. Complete gross resection has been established as an independent predictor during primary [6], interval [7,8,9] and delayed [10,11] debulking surgery, showing that the timing and cycles of systematic therapy are not as important as the absence of macroscopic residual disease after cytoreduction. Several parameters like age, performance status, the presence of ascites and the histological type of the tumor have been recognized as independent prognostic factors over the years [12,13,14,15]. Since the first report in 1996 [16] that the BRCA mutation in ovarian cancer offers improved survival, factors such as the BReast CAncer gene (BRCA) and homologous recombination deficiency (HRD) status, favorable ELIMination rate constant K (KELIM) score, and treatment with poly (ADP-ribose) polymerase inhibitors (PARPis) have been acknowledged as strong predictors for ovarian cancer patients’ survival [17,18,19,20]. Specifically, germline BRCA1/2 mutations represent the strongest known genetic risk factors for epithelial ovarian cancer, being identified in approximately 6–15%. BRCA1/2 status has important prognostic and counselling implications, as carriers demonstrate enhanced sensitivity to platinum-based chemotherapy. This increased chemosensitivity translates into improved survival outcomes, despite the fact that epithelial ovarian cancer in BRCA1/2 carriers is often diagnosed at a more advanced stage [21]. Recently, real-world data from the SUROVA study on the total postoperative complication rate during cytoreduction has been highlighted as a possible independent prognostic factor for survival [22]. However, these results should be interpreted with caution, mainly because of the study’s retrospective design.

Debulking surgery and systematic platinum-based chemotherapy, either in the adjuvant or neoadjuvant setting, are the cornerstones of advanced ovarian cancer treatment. However, due to its typical peritoneal spread, the concept of administering heated chemotherapy directly on the peritoneal surfaces, in order to eradicate the microscopic residual disease, has been investigated as an alternative [23,24]. Hyperthermic intraperitoneal chemotherapy (HIPEC) for recurrent ovarian cancer was first indicated by Sugarbaker [25]. Over the years, different techniques and chemotherapy regimen protocols have been used in HIPEC, resulting in heterogeneous data in the literature. Over the last few decades, HIPEC has been studied during primary (PDS), interval (IDS) and secondary debulking surgery (SDS) with contradictory findings, mainly from retrospective studies. Currently, HIPEC in IDS has shown the most promising results after the OVHIPEC-1 randomized controlled trial (RCT) [26], where HIPEC showed a statistically significant benefit in progression-free (PFS) and overall survival (OS), with acceptable morbidity. However, robust and high-quality data about the primary and recurrent settings are lacking, and the use of HIPEC outside the OVHIPEC-1-like interval debulking setting should still be considered investigational and cannot yet be regarded as supported by level-1 evidence. This is verified by two recent meta-analyses [27,28], where a significant survival benefit was only found in IDS. Based on the results from the SOC-1 [29] and DESKTOP III [30] trials, showing improved survival rates after SDS, and the results from the CHIPOR trial [31] and a recent systematic review [32], including not only RCTs, identifying that the addition of HIPEC to SDS gives a survival benefit, further investigation with RCTs on SDS plus HIPEC is warranted.

Hence, all the abovementioned predictors may not have the same impact in the setting of HIPEC because minimal residual disease is expected to be effectively targeted by intraperitoneal chemotherapy. The aim of this study was to identify independent prognostic factors affecting the survival rates, morbidity and recurrence patterns of advanced ovarian cancer patients in the primary setting with or without HIPEC from two certified centers. The ESPSO-certified center exclusively offered HIPEC after CRS to all patients who were included in the study, while the ESGO-certified center offered only CRS to all women with advanced ovarian cancer.

## 2. Materials and Methods

### 2.1. Study Characteristics

This multicenter retrospective study was conducted in two centers from 1 January 2015 until 31 December 2019. The first was the Surgical Oncology Department, Peritoneal Surface Malignancy Program “Kyanous Stavros” Clinic, certified by the European School of Peritoneal Surface Oncology (ESPSO), and the second was the Gynecological Oncology Unit of the 1st Department of Obstetrics & Gynecology, AUTh, “Papageorgiou” General Hospital, certified by the European Society of Gynaecological Oncology (ESGO) for Advanced Ovarian Cancer Surgery. The medical records of 333 consecutive patients with ovarian cancer treated during this period were retrieved and reviewed. Written approval was received from the Institutional Review Board (Νο. 11622, 6 March 2025).

### 2.2. Patients

Inclusion criteria:

- Patient ages between 16 and 90 years old.
- Histological confirmation of advanced epithelial ovarian cancer.
- Cytoreductive surgery in either of the two abovementioned centers.

Exclusion criteria:

- Synchronous neoplasia at the time of the diagnosis.
- Pregnancy.
- Missing important survival data.

After screening for the inclusion and exclusion criteria that were set for this study, 50 women were excluded due to early-stage disease. Moreover, 15 women were excluded because of synchronous neoplasia and 3 due to missing important survival data. Hence, finally, 265 women with advanced ovarian cancer were identified as eligible for further analysis.

Preoperatively, all patients were examined for normal renal function (blood urea level < 50 mg/dL, creatinine level < 1.5 mg/dL), normal white blood cell count (>4000) and platelets (>100.000), normal liver function tests, and satisfactory cardiopulmonary function. In order to assess resectability, all patients underwent pelvic magnetic resonance imaging (MRI) and computed tomography (CT) of the thorax, upper abdomen and retroperitoneum, and the resectability after neoadjuvant chemotherapy (NACT) was evaluated with the Response Evaluation Criteria in Solid Tumors (RECIST) [33]. In inconclusive cases, diagnostic laparoscopy was performed based on each patient’s individual characteristics, either in diagnosis or after 3 cycles of NACT. The extent of previous surgery was assessed using the prior surgery score (PSS). PSS-0 indicated that no prior surgery had been performed. Surgery in one abdominopelvic region or diagnostic laparoscopy was indicated as PSS-1, surgery in 2–5 abdominopelvic regions (mainly in the pelvis) as PSS-2, and in more than 5 regions as PSS-3. International Federation of Obstetrics and Gynecology (FIGO) stages III–IV were evaluated by imaging and confirmed at the beginning of laparotomy. The extent of peritoneal dissemination was assessed using the peritoneal cancer index (PCI).

All patients underwent cytoreductive surgery (CRS) either in the primary or interval setting, and six cycles of systematic chemotherapy were administered to the entire population. Both centers followed the same surgical protocol with radical resections in order to achieve complete gross resection, according to the ESGO guidelines [34] and PSOGI consensus [35]. However, only lymphadenectomy was left to the surgeon’s preference (removal of bulky nodes or systematic lymphadenectomy). Furthermore, the same criteria were used for the administration of systematic chemotherapy and the choice between primary CRS or NACT, with the only difference being the addition of HIPEC perfusion. HIPEC was integrated into CRS in patients of the ESPSO-certified center, while CRS alone was offered at the ESGO-certified center.

HIPEC was performed using the open abdominal (Coliseum) technique, according to details that have been previously described [36]. However, it is important to state that the HIPEC regimen protocol used in this study differs significantly from the ones from the OVHIPEC-1 trial and the NCCN [37] or ASCO [38] guidelines, where only monotherapy with cisplatin is recommended, but it is in agreement with PSOGI consensus [35], which encourages investigational bidirectional strategies. All operations were performed by two senior surgeons, one at each institution. Concerning retroperitoneal lymph node dissection, two categories were formed: systematic pelvic and para-aortic lymphadenectomy (up to the left renal vein) and selective lymphadenectomy, including the removal of enlarged/bulky nodes or omission of lymphadenectomy. Residual disease (RD) after cytoreduction was calculated with the completeness of the cytoreduction (CC) score: CC-0 for complete gross resection, CC-1 for RD ≤ 2.5 mm, CC-2 for RD > 2.5 mm and ≤2.5 cm, and CC-3 for RD ≥ 2.5 cm. In terms of recurrence patterns, local relapse was considered in the pelvis and distant in the remaining abdominal cavity or extra-abdominal.

### 2.3. Data Collection

Data were collected during a period of 30 days through medical records from the online registries of the two centers. In order to avoid inconsistencies among different dates of data collection and between centers, a uniform data collection sheet (Excel file) was used during the retrospective mining of the patients’ data. The data sheet included the following information:

- Patient’s hospital identification number.
- Patient’s age.
- European Cooperative Oncology Group (ECOG) performance status.
- American Society of Anesthesiologists Physical Status Classification System (ASA) score.
- Prior surgery score (PSS).
- Timing of cytoreduction: Primary vs. interval.
- Lymphadenectomy.
- Peritoneal cancer index (PCI).
- Intraoperative blood loss.
- Histological subtypes.
- Tumor grade.
- HIPEC perfusion.
- Completeness of cytoreduction (CC) score.
- Clavien–Dindo classification for postoperative complications.
- Hospital stay.
- Site of recurrence.
- Time-related data:
  ○ Date of surgery.
  ○ Date of recurrence.
  ○ Date of last follow-up or death.

### 2.4. Statistical Analysis

All analyses were performed using the R software, version 2025.09.0+387. For categorical variables, descriptive statistics were expressed as absolute frequencies and percentages. For continuous variables, central tendency (mean, median) and dispersion [interquartile range (IQR), standard deviation (SD)] were calculated. Cox and logistic regression for univariate and multivariate analysis were used. Progression-free survival (PFS) was defined as the time interval between the date of surgery and the date of first recurrence or disease progression, while overall survival (OS) was defined as the time interval from surgery to the date of death or last follow-up. PFS and OS analyses were performed using Kaplan–Meier curves. A *p*-value of <0.05 was considered statistically significant.

## 3. Results

This multicenter retrospective analysis initially included 333 patients with ovarian cancer. After screening for the inclusion and exclusion criteria, 265 patients with advanced ovarian cancer were eligible for further analysis. Patients underwent either CRS alone at the Gynecological Oncology Unit of the 1st Department of Obstetrics & Gynecology, AUTh, “Papageorgiou” General Hospital, or CRS plus HIPEC at the Surgical Department of Peritoneal Surface Malignancy Program, “Kyanous Stavros” Clinic, during the period of study.

The baseline characteristics of the patients are outlined in Table 1. The population of this study consisted of women with mild comorbidities, a good performance status, and a mean age of 61 years old, while the majority (75%) did not undergo a prior immediately preceding laparotomy. Primary cytoreduction was offered to 62%, with HIPEC perfusion in almost 40% (only in the ESPSO center), while selective lymph node dissection or omission of lymphadenectomy was performed in 60% of the population of this cohort. NACT followed by interval CRS was offered to 100 patients (approximately 38%). The majority of patients had serous high-grade epithelial ovarian cancer with high tumor load and dissemination in the peritoneal cavity (median PCI: 10). Concerning postoperative complications, major events occurred only in 16% of the population, with a median hospitalization of 9 days. Complete gross or near-complete resection (CC-0/1) was as high as 85%, while the majority of relapses (82%) were distant.

Furthermore, we investigated the possible prognostic factors related to major morbidity and recurrence patterns, using univariable and multivariable analysis, including all relevant parameters that were collected for this cohort, to avoid the effect of confounders.

Initially, a logistic regression analysis for the site of recurrence (local vs. distant) was performed. In the univariable analysis, a statistical significance was observed in four factors: ECOG performance status, prior surgical score, HIPEC perfusion, and lymphadenectomy. Continuing with the multivariable analysis, factors with a *p*-value < 0.2 were included. Interestingly, prior surgical score was identified as the only independent prognostic factor for the site of recurrence in advanced ovarian cancer patients. Specifically, patients with a PPS-2 had 13.5-fold higher odds of developing a local relapse, compared to those with PSS-0 (OR: 13.54, 95% CI: 2.51, 73.08, *p*-value = 0.002). The aforementioned data are detailed in Table 2.

Moreover, a logistic regression analysis for major postoperative complications was performed by using the Clavien–Dindo classification. In the univariable analysis, a statistical significance was observed in intraoperative blood loss and PCI. Continuing with the multivariable analysis, factors with a *p*-value < 0.2 were included. HIPEC perfusion, intraoperative blood loss and PCI were identified as independent prognostic factors for major postoperative complications in advanced ovarian cancer patients. Specifically, patients who were offered HIPEC perfusion had 3.5-fold higher odds of developing major postoperative complications (≥grade III) (OR: 3.58, 95% CI: 1.04, 12.29, *p*-value = 0.043). For each 1-point increase in the PCI, the odds of a major postoperative complications were almost 2-fold higher (OR: 1.86, 95% CI: 1.18, 2.93, *p*-value = 0.007), and for each 500 mL of intraoperative blood loss, the odds of major postoperative complications were increased by 65% (OR: 1.001, 95% CI: 1.00, 1.002, *p*-value = 0.019). The aforementioned data are detailed in Table 3.

**Figure 1:**
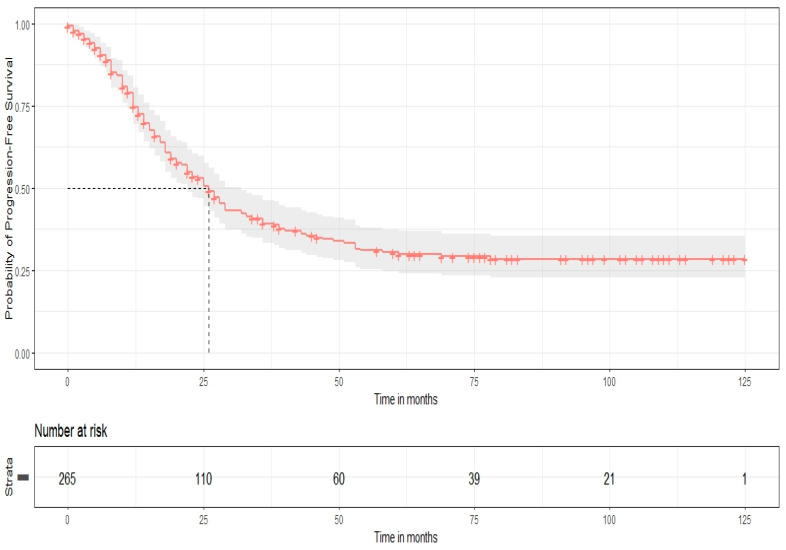
Progression-free survival (Kaplan–Meier curve).

**Figure 2:**
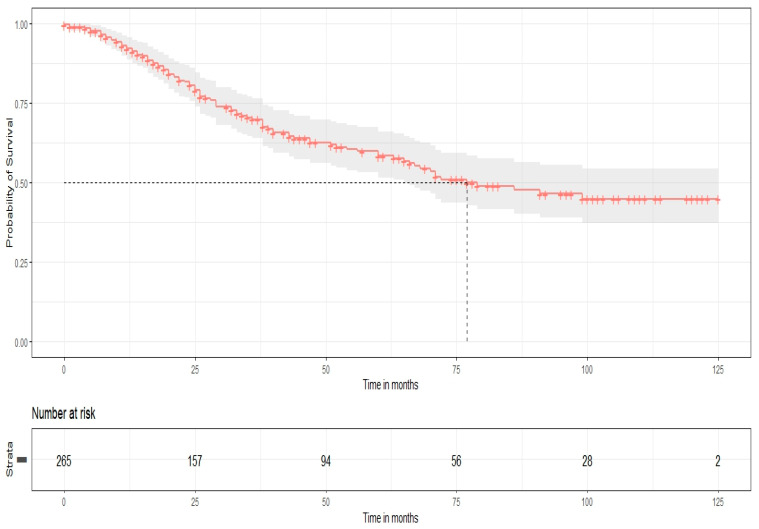
Overall survival (Kaplan–Meier curve).

**Figure 3:**
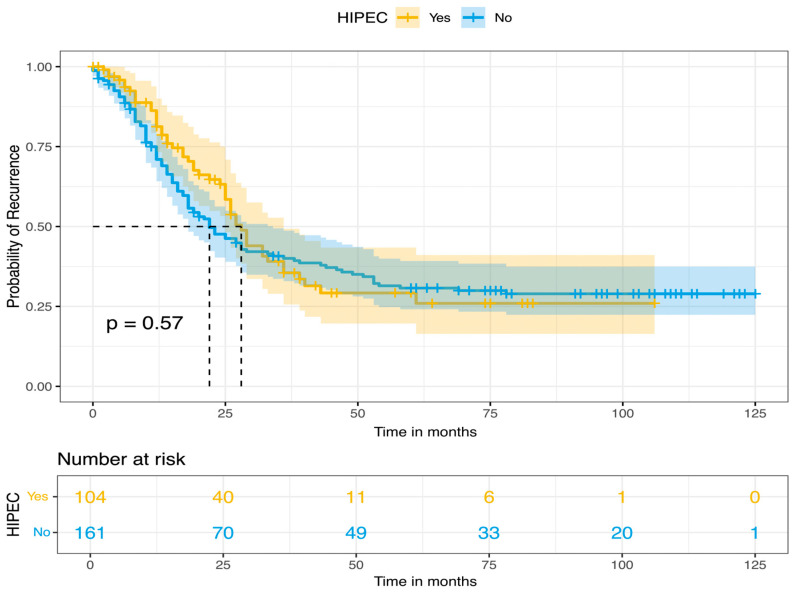
Progression-free survival, based on HIPEC perfusion (Kaplan–Meier curve).

**Figure 4:**
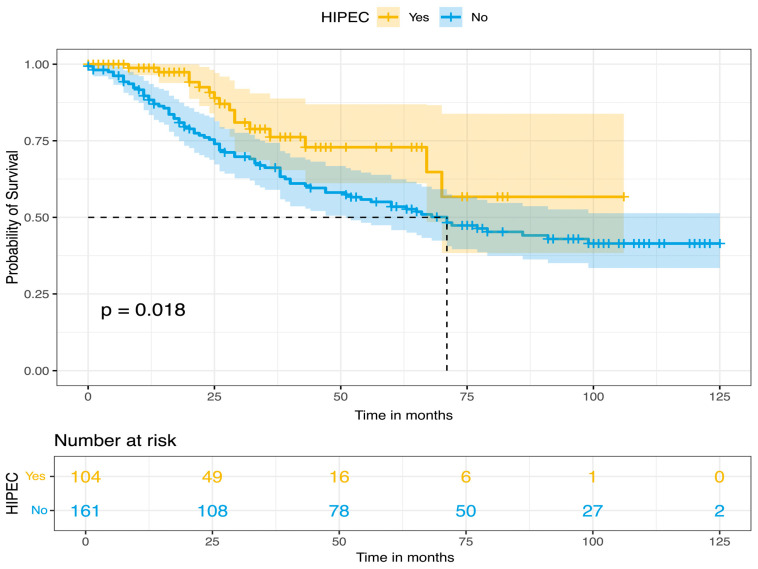
Overall survival, based on HIPEC perfusion (Kaplan–Meier curve).

All patients were closely monitored with routine review visits, every 4 months for the first year, every 6 months until 5 years of follow-up, and once a year thereafter. The median follow-up time was 34 months. Survival outcomes were estimated by using Kaplan–Meier survival curves. For the whole cohort’s population, the median PFS and OS were 26 and 77 months, respectively, while the 5-year survival rates for PFS and OS were 30% and 58%, respectively. Furthermore, based on HIPEC perfusion, Kaplan–Meier curves for PFS and OS were constructed. In the long-rank test, no statistical significance was observed for PFS (*p*-value = 0.57), but there was a significantly improved OS for patients with CRS plus HIPEC (*p* = 0.018). These results are presented in Figure 1, Figure 2, Figure 3 and Figure 4.

In order to investigate possible prognostic factors affecting the survival rates of the patients, a univariable and multivariable Cox regression analysis was conducted. Firstly, for the risk of recurrence or disease progression, the univariable analysis identified a statistical significance for age, ECOG performance status, timing of cytoreduction, histological subtypes, tumor grade, lymphadenectomy, ≥grade III postoperative complications, PCI, and completeness of cytoreduction. In the multivariable analysis, factors with a *p*-value < 0.2 were included. Only lymphadenectomy and histological subtypes were identified as independent prognostic factors for PFS in advanced ovarian cancer patients. Specifically, patients who underwent selective lymphadenectomy had approximately double the risk of recurrence of disease progression, compared to those with systematic (HR: 1.81, 95% CI: 1.22, 2.69, *p*-value = 0.003). Likewise, the patients with non-serous histology had a 2-fold higher risk of recurrence (HR: 2.12, 95% CI: 1.18, 3.84, *p*-value = 0.013). The aforementioned data are presented in detail in Table 4.

Secondly, for the risk of death, the univariable analysis identified statistical significance for age, ASA score, ECOG performance status, timing of cytoreduction, HIPEC perfusion, histological subtypes, tumor grade, lymphadenectomy, PCI, and completeness of cytoreduction. In the multivariable analysis, factors with a *p*-value < 0.2 were only included. Completeness of cytoreduction, lymphadenectomy and histological subtypes were identified as independent prognostic factors for OS. Specifically, patients with macroscopical gross residual tumor disease (CC-3) had an approximately 3.5-fold higher risk of death compared to patients with complete gross resection (HR: 3.32, 95% CI: 1.34, 8.25, *p*-value = 0.010), and when selective lymphadenectomy was offered, the risk of death almost doubled, compared to systematic lymphadenectomy (HR: 1.88, 95% CI: 1.04, 3.43, *p*-value = 0.038). In addition, patients with non-serous histology had a 2.5-fold higher risk of death (HR: 2.54, 95% CI: 1.13, 5.71, *p*-value = 0.024). The aforementioned data are summarized in Table 5.

## 4. Discussion

The primary objective of our study was to investigate possible independent prognostic factors affecting survival rates, recurrence patterns and postoperative morbidity in primary diagnosed advanced-stage ovarian cancer patients in the HIPEC setting. We retrospectively reviewed 265 patients who underwent either CRS alone or CRS plus HIPEC and further analyzed them. The majority of patients (85.6%) underwent optimal cytoreduction with CC-0/1. Patients who had CC-1 with an RD ≤ 2.5 mm (36.2%) are of special interest because, from a biological standpoint, HIPEC is intended to eradicate microscopic or minimal residual disease. So, these patients are those most likely to benefit from intraperitoneal chemotherapy because CC-1 is generally regarded as the threshold for offering HIPEC after cytoreduction.

Concerning the site of recurrence, patients with PSS-2 have a much higher risk of loco-regional relapse compared to those with PSS-0. Multivariable analysis for major postoperative complications, calculated with Clavien–Dindo classification, revealed HIPEC perfusion, PCI, and intraoperative blood loss as independent prognostic factors. However, the total percentage of ≥grade III complications (16.6%) in this cohort was acceptable and comparable to previous publications. Therefore, these results on the association between HIPEC and major postoperative complications should be interpreted with caution because of the different regimen protocol used in this study. For the survival rates, selective lymphadenectomy and non-serous histology were identified as independent prognostic factors for PFS and OS, with a higher risk of recurrence and death. Residual disease > 2.5 mm was statistically significant in the univariable analysis for both PFS and OS, but only macroscopic gross residual disease (CC-3) was an independent negative prognostic factor for OS alone. Furthermore, Kaplan–Meier survival analysis showed that CRS plus HIPEC in the primary and interval setting had a statistically better OS, compared to CRS alone, but HIPEC lost its statistical significance for the risk of death in the multivariable analysis. Therefore, these survival analysis results should be interpreted with caution because of selection bias from a retrospective, non-randomized design and center-specific policy from the different perfusion regimen protocol that was used and the different settings (primary and interval) that were pooled together in the survival analysis. The inclusion of patients undergoing CC-2 (9.2%) and CC-3 (5.3%) surgery reflects the inability of the imaging modalities to identify that the 14.4% of the patients should have been excluded from surgery.

To our knowledge, there are only a few studies in the literature that have investigated independent prognostic factors in advanced ovarian cancer in the era of HIPEC. Two studies investigated HIPEC after secondary cytoreduction. The first publication was a retrospective multicenter study from France [39] that included 42 patients and found that HIPEC is an independent prognostic factor for OS. However, the second one, a retrospective single-center study from Brazil [40], included 79 patients and did not confirm the abovementioned results, showing that HIPEC is not an independent prognostic factor for the risk of death. Moreover, a retrospective single-center study from South Korea [41] included 123 patients and showed that HIPEC is an independent prognostic factor for the risk of recurrence or disease progression, but no data about the other covariates in the univariable and multivariable analyses were produced. The most recent study from Karanikas et al. [36] included 151 patients and showed that HIPEC was an independent prognostic factor not only for OS but also for DFS, which is in contradiction to our results. Possible explanations for this difference are the much larger sample size (*n* = 265) and the fact that CRS-alone patients underwent surgery at an ESGO-certified center, and CRS plus HIPEC patients underwent surgery at an ESPSO-certified center, providing high-quality treatment for both groups.

Last but not least, our finding that prior surgery in a non-certified, low-volume center has a significant detrimental impact on loco-regional relapse is in agreement with previous publications suggesting that patients operated on by gynecological oncologists in high-volume certified centers have an improved survival probability [42,43]. Therefore, our results highlight the need for treatment in specialized centers, while diagnostic laparoscopy (PPS-1) for histological confirmation and complete resectability assessment does not alter recurrence patterns. In addition, in our study, there was a high rate (almost 40%) of systematic pelvic and paraaortic lymphadenectomy for advanced ovarian cancer, which is not in agreement with the current ESGO guidelines, where lymphadenectomy is omitted. Furthermore, our finding that the removal of bulky lymph nodes or the omission of lymphadenectomy during CRS is an independent prognostic factor for worse PFS and OS is in complete contradiction with the results of the Lymphadenectomy in Ovarian Neoplasms (LION) study [44], which showed no benefit in PFS or OS for systematic lymphadenectomy in advance-stage ovarian cancer, leading to a gradual change in everyday clinical practice from 2017 to 2019. So, the high rate of systematic lymphadenectomy is explained by the time period of our study (2015 to 2019). On the other hand, our results are in agreement with a study from the AGO Ovarian Cancer Study (AGO-OVAR) group [45], which performed an exploratory analysis of three prospective randomized trials and showed that systematic lymphadenectomy might offer survival benefits when complete gross resection is achieved in advanced ovarian cancer. However, none of the abovementioned studies included patients who underwent HIPEC perfusion. So, our results suggest that possible microscopic retroperitoneal lymph node disease plays an important role in survival rates when HIPEC perfusion is performed for microscopic peritoneal surface disease. Moreover, our finding that CRS plus HIPEC leads to a significantly improved OS, especially when 60% of the population underwent primary cytoreduction, further supports the beneficial role of HIPEC in the primary setting, while high-quality data from the OVHIPEC-2 trial are awaited [46].

The strengths of this study included the large sample size and the long follow-up period. Adjustment for clinically relevant covariates through multivariable modeling reduced bias and supported the robustness of the main findings in the HIPEC setting. Furthermore, both centers of the study are certified by ESGO or ESPSO, ensuring a high level of quality indicators and surgical proficiency. This is also confirmed by the high (85.6%) complete gross or optimal resection rate. On the other hand, the main limitation of our study is its retrospective nature. Furthermore, the different HIPEC perfusion regimen protocols, from the classic proposed one, might affect the generalizability of the results, and the fact that HIPEC was offered at only one of the two centers of the study could also be a possible bias. Additionally, systemic treatment heterogeneity over time was not assessed, and data on bevacizumab and PARPi maintenance therapies and BRCA/HRD status were not collected. These are possible biases because they could influence survival rates.

The principal contribution of this study to the literature is the addition of real-world, high-volume setting data on CRS plus HIPEC, which complement RCT-driven evidence from OVHIPEC trials. Future large prospective studies are needed in order to establish the aforementioned independent prognostic factors, as well as randomized controlled trials on the role of systematic lymphadenectomy with HIPEC perfusion in advanced ovarian cancer.

## 5. Conclusions

CRS plus HIPEC improves OS in advanced ovarian cancer patients. These results should be viewed cautiously because of possible selection bias from the different regimen perfusion protocols and pooled cases that were treated primarily and after NACT. Furthermore, HIPEC was not identified as an independent prognostic factor affecting survival rates, but it was for major postoperative complications. However, this should be interpreted with caution because different regimen perfusion protocols might affect complication rates. Systematic lymphadenectomy and serous histology were found to be independent prognostic factors, associated with improved PFS and OS, while macroscopic gross residual disease was only associated with OS. Last but not least, patients with an immediately preceding laparotomy in the pelvis had a significantly higher chance of loco-regional relapse.

## Figures and Tables

**Table 1:** Baseline characteristics.

		Number of Patients (N)	Percentage (%)
Age (Years)		mean: 61	SD: 12
ASA score			
	I	199	75.1
	II	49	18.5
	III	17	6.4
ECOG performance status			
	0	154	58.1
	1	82	30.9
	2	29	10.9
Prior surgical score			
	PSS-0	130	49.1
	PSS-1	67	25.3
	PSS-2	49	18.5
	PSS-3	19	7.2
Timing of cytoreduction			
	primary	165	62.3
	interval	100	37.7
HIPEC			
	no	161	60.8
	yes	104	39.2
Histological subtypes			
	serous	206	77.7
	endometrioid	25	9.4
	clear cell	13	4.9
	mucinous	13	4.9
	other	8	3
Tumor grade			
	low	40	15.1
	high	225	84.9
Lymphadenectomy			
	systematic	104	39.2
	selective	161	60.8
Intraoperative blood loss (mL)		median: 200	IQR: 100–500
PCI		median: 10	IQR: 5–17
Clavien–Dindo classification			
	grade I–II	221	83.4
	≥grade III	44	16.6
Hospital stay (days)		median: 9	IQR: 7–12
Completeness of cytoreduction			
	CC-0	131	49.4
	CC-1	96	36.2
	CC-2	24	9.1
	CC-3	14	5.3
Recurrence site			
	local	37	18.2
	distant	166	81.8

**Table 2:** Logistic regression analysis for recurrence site.

		Univariate			Multivariate		
		OR	95% CI	*p*-Value	OR	95% CI	*p*-Value
Age (years)		1.02	0.99, 1.06	0.238			
ASA score							
	I	1	1	1	1	1	1
	II	0.37	0.12, 1.14	0.084	1.43	0.26, 7.85	0.682
	III	0.28	0.03, 2.28	0.233	0.95	0.07, 12.17	0.969
ECOG performance status							
	0	1	1	1	1	1	1
	1	0.21	0.08, 0.59	**0.003**	0.82	0.15, 4.33	0.813
	2	0.19	0.04, 0.87	**0.032**	0.84	0.07, 9.41	0.885
Prior surgical score							
	PSS-0	1	1	1	1	1	1
	PSS-1	1.98	0.70, 5.56	0.197	1.89	0.61, 5.90	0.271
	PSS-2	16.25	5.29, 49.92	**<0.001**	13.54	2.51, 73.08	**0.002**
	PSS-3	5.42	1.25, 23.39	**0.024**	4.73	0.79, 28.32	0.089
Timing of cytoreduction							
	primary	1	1	1	1	1	1
	interval	2.12	0.98, 4.57	0.055	1.33	0.47, 3.75	0.590
HIPEC perfusion							
	no	1	1	1	1	1	1
	yes	8.18	3.57, 18.75	**<0.001**	1.91	0.50, 7.29	0.346
Histological subtypes							
	serous	1	1	1			
	non-serous	1.13	0.35, 3.68	0.836			
Tumor grade							
	low	1	1	1			
	high	1.07	0.29, 4.11	0.922			
Lymphadenectomy							
	systematic	1	1	1	1	1	1
	selective	0.36	0.16, 0.78	**0.009**	0.36	0.12, 1.07	0.066
PCI		1.01	0.96, 1.07	0.595			
Completeness of cytoreduction							
	CC-0	1	1	1			
	CC-1	0.91	0.40, 2.07	0.825			
	CC-2	0.37	0.08, 1.76	0.210			
	CC-3	2.36	0.57, 9.74	0.237			

Boldface text indicates statistical significance (*p* < 0.05).

**Table 3:** Logistic regression for major postoperative complications.

		Univariate			Multivariate		
		OR	95% CI	*p*-Value	OR	95% CI	*p*-Value
Age (years)		1.00	0.97, 1.02	0.796			
ASA score							
	I	1	1	1			
	II	0.61	0.24, 1.54	0.297			
	III	0.27	0.04, 2.13	0.216			
ECOG performance status							
	0	1	1	1	1	1	1
	1	0.57	0.27, 1.24	0.159	0.79	0.28, 2.27	0.661
	2	0.66	0.21, 2.04	0.473	0.66	0.16, 2.71	0.568
Prior surgical score							
	PSS-0	1	1	1	1	1	1
	PSS-1	0.52	0.21, 1.27	0.149	0.36	0.13, 1.00	0.051
	PSS-2	0.99	0.43, 2.32	0.988	0.41	0.13, 1.33	0.137
	PSS-3	1.18	0.36, 3.87	0.787	0.60	0.13, 2.72	0.510
Timing of cytoreduction							
	primary	1	1	1			
	interval	0.73	0.37, 1.46	0.376			
HIPEC perfusion							
	no	1	1	1	1	1	1
	yes	1.70	0.88, 3.25	0.112	3.58	1.04, 12.29	**0.043**
Histological subtypes							
	serous	1	1	1	1	1	1
	non-serous	2.00	0.80, 4.99	0.138	0.87	0.29, 2.57	0.794
Tumor grade							
	low	1	1	1	1	1	1
	high	4.13	0.96, 17.84	0.057	2.72	0.53, 14.00	0.233
Lymphadenectomy							
	systematic	1	1	1	1	1	1
	selective	1.90	0.93, 3.89	0.078	2.29	0.92, 5.70	0.074
Intraoperative blood loss (mL)		1.001	1.00, 1.002	**0.032**	1.001	1.00, 1.002	**0.019**
PCI		1.07	1.03, 1.12	**<0.001**	1.86	1.18, 2.93	**0.007**
Completeness of cytoreduction							
	CC-0	1	1	1			
	CC-1	0.83	0.41, 1.67	0.825			
	CC-2	0.89	0.28, 2.85	0.847			
	CC-3	0.34	0.04, 2.75	0.314			

Boldface text indicates statistical significance (*p* < 0.05).

**Table 4:** Cox regression for recurrence or disease progression.

		Univariate			Multivariate		
		HR	95% CI	*p*-Value	HR	95% CI	*p*-Value
Age (years)		1.02	1.004, 1.03	**0.011**	1.01	0.99, 1.02	0.221
ASA score							
	I	1	1	1	1	1	1
	II	1.29	0.88, 1.89	0.197	0.81	0.45, 1.45	0.480
	III	0.91	0.47, 1.73	0.766	1.04	0.44, 2.28	0.915
ECOG performance status							
	0	1	1	1	1	1	1
	1	1.05	0.74, 1.49	0.783	1.06	0.68, 1.66	0.786
	2	1.88	1.19, 2.99	**0.007**	1.18	0.53, 2.64	0.684
Prior surgical score							
	PSS-0	1	1	1			
	PSS-1	1.12	0.78, 1.62	0.537			
	PSS-2	1.04	0.64, 1.67	0.884			
	PSS-3	1.12	0.58, 2.16	0.747			
Timing of cytoreduction							
	primary	1	1	1	1	1	1
	interval	1.94	1.41, 2.66	**<0.001**	1.41	0.98, 2.03	0.064
HIPEC perfusion							
	no	1	1	1			
	yes	0.91	0.65, 1.27	0.572			
Histological subtypes							
	serous	1	1	1	1	1	1
	non-serous	3.66	2.23, 6.02	**<0.001**	2.12	1.18, 3.84	**0.013**
Tumor grade							
	low	1	1	1	1	1	1
	high	3.43	1.90, 6.20	**<0.001**	1.57	0.79, 3.11	0.197
Lymphadenectomy							
	systematic	1	1	1	1	1	1
	selective	2.55	1.80, 3.60	**<0.001**	1.81	1.22, 2.69	**0.003**
Clavien–Dindo classification							
	grade I–II	1	1	1	1	1	1
	≥grade III	1.73	1.13, 2.64	**0.011**	1.29	0.82, 2.04	0.270
PCI		1.05	1.03, 1.07	**<0.001**	1.02	0.99, 1.05	0.131
Completeness of cytoreduction							
	CC-0	1	1	1	1	1	1
	CC-1	1.05	0.74, 1.48	0.801	1.00	0.65, 1.55	0.984
	CC-2	2.13	1.27, 3.57	**0.004**	1.23	0.60, 2.51	0.579
	CC-3	2.91	1.44, 5.86	**0.003**	2.18	0.96, 4.97	0.064

Boldface text indicates statistical significance (*p* < 0.05).

**Table 5:** Cox regression for death.

		Univariate			Multivariate		
		HR	95% CI	*p*-Value	HR	95% CI	*p*-Value
Age (years)		1.02	1.002, 1.04	**0.031**	1.01	0.99, 1.03	0.361
ASA score							
	I	1	1	1	1	1	1
	II	1.69	1.07, 2.68	**0.025**	0.97	0.50, 1.89	0.927
	III	1.54	0.73, 3.21	0.256	1.59	0.63, 4.03	0.328
ECOG performance status							
	0	1	1	1	1	1	1
	1	1.49	0.95, 2.32	0.084	1.08	0.56, 2.08	0.812
	2	2.94	1.68, 5.15	**<0.001**	1.34	0.47, 3.79	0.581
Prior surgical score							
	PSS-0	1	1	1			
	PSS-1	1.08	0.69, 1.69	0.746			
	PSS-2	0.78	0.37, 1.64	0.505			
	PSS-3	0.84	0.34, 2.10	0.709			
Timing of cytoreduction							
	primary	1	1	1	1	1	1
	interval	1.82	1.22, 2.73	**0.004**	1.09	0.68, 1.72	0.723
HIPEC perfusion							
	no	1	1	1	1	1	1
	yes	0.52	0.30, 0.90	**0.02**	0.71	0.34, 1.47	0.351
Histological subtypes							
	serous	1	1	1	1	1	1
	non-serous	4.21	2.21, 8.41	**<0.001**	2.54	1.13, 5.71	**0.024**
Tumor grade							
	low	1	1	1	1	1	1
	high	3.89	1.70, 8.90	**<0.001**	1.70	0.67, 4.35	0.266
Lymphadenectomy							
	systematic	1	1	1	1	1	1
	selective	3.31	2.02, 5.42	**<0.001**	1.88	1.04, 3.43	**0.038**
Clavien–Dindo classification							
	grade I–II	1	1	1	1	1	1
	≥grade III	1.51	0.84, 2.71	0.173	1.19	0.64, 2.24	0.582
PCI		1.07	1.04, 1.09	**<0.001**	1.02	0.99, 1.06	0.191
Completeness of cytoreduction							
	CC-0	1	1	1	1	1	1
	CC-1	1.34	0.84, 2.14	0.213	1.10	0.62, 1.95	0.740
	CC-2	3.07	1.65, 5.70	**<0.001**	1.42	0.61, 3.27	0.414
	CC-3	5.58	2.68, 11.63	**<0.001**	3.32	1.34, 8.25	**0.010**

Boldface text indicates statistical significance (*p* < 0.05).

## Data Availability

In accordance with the journal’s guidelines, the data presented in this study are available upon request from the corresponding author for the reproducibility of this study if such is requested.

## Abbreviations

The following abbreviations are used in this manuscript:

CRS	Cytoreductive surgery
HIPEC	Hyperthermic intraperitoneal chemotherapy
NACT	Neoadjuvant chemotherapy
ESGO	European Society of Gynaecological Oncology
ESPSO	European School of Peritoneal Surface Oncology
PARPi	Poly (ADP-ribose) polymerase inhibitors
BRCA	BReast CAncer gene
HRD	Homologous recombination deficiency
KELIM	ELIMination rate constant K
PDS	Primary debulking surgery
IDS	Interval debulking surgery
SDS	Secondary debulking surgery
PFS	Progression-free survival
OS	Overall survival
RCTs	Randomized controlled trials
MRI	Pelvic magnetic resonance imaging
CT	Computed tomography
PSS	Prior surgery score
FIGO	International Federation of Obstetrics and Gynecology
PCI	Peritoneal cancer index
RD	Residual disease
CC	Completeness of cytoreduction
ECOG	European Cooperative Oncology Group
ASA	American Society of Anesthesiologists Physical Status Classification System
IQR	Interquartile range
SD	Standard deviation
OR	Odds ratio
HR	Hazard ratio
AGO-OVAR	AGO ovarian cancer study
LION	Lymphadenectomy in Ovarian Neoplasms
